# *In situ* performance and stability tests of large-area flexible polymer solar cells in the 35-km stratospheric environment

**DOI:** 10.1093/nsr/nwac285

**Published:** 2022-12-15

**Authors:** Zihan Xu, Guoning Xu, Qun Luo, Yunfei Han, Yu Tang, Ying Miao, Yongxiang Li, Jian Qin, Jingbo Guo, Wusong Zha, Chao Gong, Kun Lu, Jianqi Zhang, Zhixiang Wei, Rong Cai, Yanchu Yang, Zhaojie Li, Chang-Qi Ma

**Affiliations:** i-Lab & Printable Electronic Center, Suzhou Institute of Nano-Tech and Nano-Bionics, Chinese Academy of Sciences, Suzhou 215123, China; School of Nano-Tech and Nano-Bionics, University of Science and Technology of China, Hefei 230027, China; Aerospace Information Research Institute, Chinese Academy of Sciences, Beijing 100094, China; University of Chinese Academy of Sciences, Beijing 100049, China; i-Lab & Printable Electronic Center, Suzhou Institute of Nano-Tech and Nano-Bionics, Chinese Academy of Sciences, Suzhou 215123, China; School of Nano-Tech and Nano-Bionics, University of Science and Technology of China, Hefei 230027, China; i-Lab & Printable Electronic Center, Suzhou Institute of Nano-Tech and Nano-Bionics, Chinese Academy of Sciences, Suzhou 215123, China; Aerospace Information Research Institute, Chinese Academy of Sciences, Beijing 100094, China; Aerospace Information Research Institute, Chinese Academy of Sciences, Beijing 100094, China; Aerospace Information Research Institute, Chinese Academy of Sciences, Beijing 100094, China; i-Lab & Printable Electronic Center, Suzhou Institute of Nano-Tech and Nano-Bionics, Chinese Academy of Sciences, Suzhou 215123, China; i-Lab & Printable Electronic Center, Suzhou Institute of Nano-Tech and Nano-Bionics, Chinese Academy of Sciences, Suzhou 215123, China; i-Lab & Printable Electronic Center, Suzhou Institute of Nano-Tech and Nano-Bionics, Chinese Academy of Sciences, Suzhou 215123, China; i-Lab & Printable Electronic Center, Suzhou Institute of Nano-Tech and Nano-Bionics, Chinese Academy of Sciences, Suzhou 215123, China; CAS Key Laboratory of Nanosystem and Hierarchical Fabrication, National Center for Nanoscience and Technology, Beijing 100190, China; CAS Key Laboratory of Nanosystem and Hierarchical Fabrication, National Center for Nanoscience and Technology, Beijing 100190, China; CAS Key Laboratory of Nanosystem and Hierarchical Fabrication, National Center for Nanoscience and Technology, Beijing 100190, China; Aerospace Information Research Institute, Chinese Academy of Sciences, Beijing 100094, China; Aerospace Information Research Institute, Chinese Academy of Sciences, Beijing 100094, China; Aerospace Information Research Institute, Chinese Academy of Sciences, Beijing 100094, China; i-Lab & Printable Electronic Center, Suzhou Institute of Nano-Tech and Nano-Bionics, Chinese Academy of Sciences, Suzhou 215123, China; School of Nano-Tech and Nano-Bionics, University of Science and Technology of China, Hefei 230027, China

**Keywords:** flexible organic solar cells, stratospheric environment, 35-km altitude, thermal cycle, reliability

## Abstract

Flexible organic solar cells (FOSCs) are one of the most promising power sources for aerospace aircraft due to their attractive advantages with high power-per-weight ratio and excellent mechanical flexibility. Understanding the performance and stability of high-performance FOSCs is essential for the further development of FOSCs for aerospace applications. In this paper, after systematic investigations on the performance of the state-of-the-art high-performance solar cells under thermal cycle and intensive UV irradiation conditions, *in situ* performance and stability tests of the solar cells in the 35 km stratospheric environment were carried out through a high-altitude balloon uploading. The encapsulated FOSCs with an area of 0.64 cm^2^ gave the highest power density of 15.26 mW/cm^2^ and an efficiency over 11%, corresponding to a power-per-weight ratio of over 3.32 kW/kg. More importantly, the cells showed stable power output during the 3-h continuous flight at 35 km and only 10% performance decay after return to the lab, suggesting promising stability of the FOSCs in the stratospheric environment.

## Teaser

Flexible organic solar cell was launched to 35 km stratosphere through a high-altitude balloon, showing a high specific power ratio of 3.32 kW/kg and very good stability.

## INTRODUCTION

Near-space aircraft and high-altitude pseudo satellites in the stratosphere have a wide application in environmental monitoring, disaster relief and mitigation, agricultural and forestry monitoring, resource exploration, and communication [1]. Since they are remote from the earth, solar energy is the best energy source for near-space aircraft and high-altitude pseudo satellites. Unlike on earth, solar panels for space application should have a high power-per-weight ratio with excellent reliability in the stratospheric environment, which is essential in reducing the overall weight of the power system and consequently increasing the payload and endurance of the aircraft. Therefore, photovoltaic technologies with high power-per-weight ratio, including ultrathin silicon solar cells [2,3] and III-V multijunction cells are mostly included for this purpose [4]. In comparison with these mature solar cell technologies, the emerging nano-thin film solar cells, including organic solar cells (OSCs) and perovskite solar cells (PeroSC) are very attractive for aerospace applications owing to the features of high power-per-weight ratio and excellent flexibility [5,6] that originate from their ultrathin layered structure. Conceptual proofs of the performance of OSC and PeroSC at stratospheric and satellite altitudes were carried out by Cardinaletti *et al*. [7], Zhu *et al*. [8] and Müller-Bushbaum *et al*. [9] using high-altitude balloons at 35 km or a rocket flight at 240 km, respectively. Although most of the cells for the space tests were rigid ITO-based small-area cells, these preliminary results have clearly shown great possibility for the space application of these emerging solar cell technologies.

With the rapid development of non-fullerene small molecule acceptors [10,11] and flexible transparent electrodes [12], flexible organic solar cells (FOSCs) have reached high performances of 17.5% [13] and 16.71% [14] for 0.062 and 1 cm^2^ cells, respectively, which are close to that of the corresponding rigid ITO-glass based cells [15]. More importantly, ultrathin OSCs with a total thickness of a few micrometers can be achieved using plastic substrates like polyethylene terephthalate (PET) [16], polydimethylsiloxane (PDMS) [5], polyethylenaphthalate (PEN) [17], polyimide (PI) [18,19] and perylene [20]. With these, the highest power-per-weight, over 33 kW/kg, was reported [6], pushing the OSCs forward to near-space applications. For use in space, the devices will suffer from extreme environments, including large temperature contrast, high vacuum, strong ultraviolet radiation, and various solar cosmic rays [21]. Regarding these, some terrestrial simulated experiments have been carried out. For example, Lee *et al.* found polymer:fullerene OSCs have good durability under five complete thermal cycles between −100 and 80°C [22]. Meanwhile, Troshin *et al.* demonstrated impressive radiation resistibility of the PCDTBT : PC_61_BM OSCs, with ∼90% of efficiency remaining when exposed to radiation at a dose of 6500 Gy, which was equivalent to 10 years of space radiation dose [23]. For stratospheric usage, the FOSCs module was tested in near-space in the frame of Optical Sensors based on CARbon materials mission (OSCAS) [7], however, the performance of the FOSC module was around 1.6%, which is much lower than the state-of-the-art PCE of the FOSCs. No further research work on the *in-situ* performance and stability test of the FOSCs in the stratosphere environment was reported.

In this work, we explored the *in situ* performance and stability of large-area FOSCs in the 35-km stratosphere environment through a high-altitude balloon. Before the *in situ* performance measurement, systematical simulation experiments proved the reliability of FOSCs under alternated temperature change and intensive UV irradiation. The *in situ* measurement results showed that FOSCs gave the highest power density of 15.26 mW/cm^2^ and efficiency of 11.16% at 35 km, which are the record power and performance of OSCs in the space environment. In addition, the FOSCs kept stable over 3-h continuous flying at 35 km. These results are of great significance for space solar cells and show a great possibility of large-area FOSCs for space usage.

## RESULTS AND DISCUSSION

### Performance and stability tests of the FOSCs in the lab

**Figure 1.**
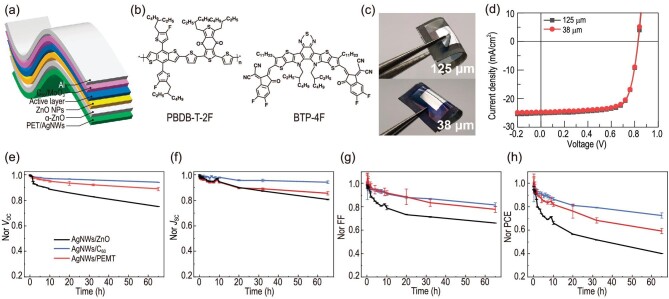
(a) Device structure, (b) the molecular structures of PBDB-T-2F and BTP-4F, (c) photographs and (d) the *J−V* characteristics of the FOSCs. (e–h) Evolution of the performance during irradiation under 365-nm UV illumination for 60 h.

FOSCs with an inverted structure of AgNWs/α-ZnO/ZnO NPs/PBDB-T-2F : BTP-4F/C_60_/MoO_3_/Al [24] with an area of 0.64 cm^2^ were fabricated using 125 or 38 μm PET as substrates (Fig. 1a). Figure 1b and c show the molecular structures of PBDB-T-2F and BTP-4F, and the photographs of the devices. The *J−V* characteristics and performance parameters of these devices are shown in Fig. 1d and Table S1. A highest efficiency of 14.61% and 15.01% was achieved for the 125- and 38-μm–thick PET substrate-based devices. These device performances were comparable to the small-area FOSCs we have reported previously [24,25], and among the highest performance of the large-area FOSCs with a PBDB-T-2F : BTP-4F photoactive layer [26–29]. This result indicated the suitability of AgNWs electrode for large-area high-performance ultrathin FOSCs. In addition, the FOSCs on 38-μm–thick substrates (Fig. S1) yielded an extremely high power-per-weight of 3.32 kW/kg, making this type of solar cell ideal for use in space.

In this work, ZnO nanoparticle (NP) was chosen as the electron transporting layer (ETL) because of its high working thickness, which could ensure total coverage of the relatively rough AgNWs by ETLs and high performance of the devices. While other polymer ETLs, i.e. PEI-, PFN-Br–, and PDINO ETL-based devices showed short-circuit or inferior device performance due to their poor coverage on the AgNWs electrode (as shown in Fig. S2). In terms of space application, the FOSCs have to withstand strong UV irradiation; we know the typical photocatalyst effect of ZnO would accelerate performance degradation during long-term illumination, specifically in the case of UV irradiation [26,30]. To solve this problem, we modified the ZnO ETLs with C_60_ and 2-phenylethylmercaptan (PEMT). Figure 1e–h shows the evolution of the performance parameter during 60-h irradiation under 365-nm UV illumination. We found all the devices showed a gradual decline of *V*_OC_, *J*_SC_, and FF under UV light illumination. The decreased *V*_OC_ and FF would be ascribed to the change in the work function of ZnO. It was found the surface potential of ZnO (Figs S3 and S4) increased by 0.24 eV during UV irradiation. The decrease in *J*_SC_ was due to the decomposition of the non-fullerene acceptor because of the photocatalyst effect of ZnO, which could be evidenced by the UV-vis absorbance spectra (Fig. S5). Compared with the pristine ZnO ETL, the devices with ZnO/C_60_ or ZnO/PEMT ETL declined at a much slower rate, fully proving that the insertion of the C_60_ derivative [10,31] or PEMT [28,30] between ZnO and the organic photoactive layer is effective in restraining the interface degradation under UV irradiation. Compared with ZnO/PEMT, the insertion of C_60_ might form a more compact barrier layer between ZnO and the organic layer, thereby leading to the slowest degradation speed. In detail, 80% of the initial efficiency remained after 60-h illumination, while only 40% of the initial efficiency remained for the pristine device (Fig. 1h). Based on these results, ZnO/C_60_ ETL was utilized as the ETL in the following work.

**Figure 2.**
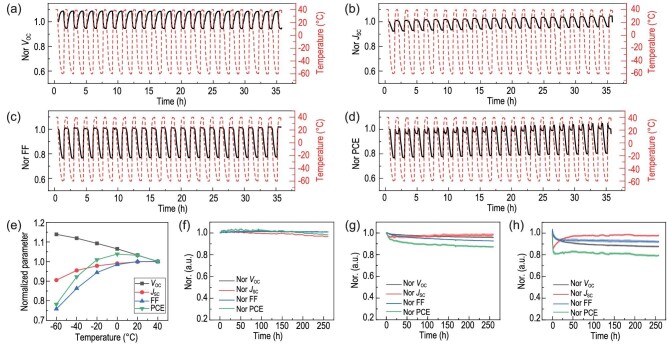
Evolution of (a) *V*_OC_, (b) *J*_SC_, (c) FF and (d) PCE during the thermal cycle. (e) Temperature-dependent device performance (normalized with values of 40^o^C). Evolution of the device performance under continuous illumination at (f) −60°C, (g) 25°C and (h) 85°C.

We know the space environment will have large temperature variations and rapid temperature changes, which would lead to the formation of cracks or delamination due to repeated material expansion and compression. Thus, the performance change under rapid thermal cycles is vital. According to the *in situ* temperature data, we found the real temperature at 35-km high altitude in daylight typically changes from −40 to 40°C (*vide infra*), which has also been reported in previous works [7,8]. Therefore, we evaluated the performance and durability of the FOSCs during thermal cycles with temperatures varying from 40 to −60°C (Fig. 2a–d). In detail, the devices were stored in the LED light source integrated climate chamber (Fig. S6), and *J−V* curves were periodically recorded under continuous illumination. As shown in Fig. 2, we found *V*_OC_ at −60^o^C was higher than that at 40^o^C, while devices *J*_SC_ and FF were lower at low temperatures. As a consequence, device efficiency at −60°C was around 80% of the efficiency of that at 40°C. Similar thermal cycle results of OSCs have been reported previously [22]. The higher *V*_OC_ at −60^o^C relative to 40^o^C would be ascribed to less material disorder [32], and lower *J*_SC_ and FF at −60°C might be due to relatively low carrier transporting [22]. Though the FOSCs showed inferior performance at low temperatures, the device remained stable during 40 thermal cycles, implying the devices could sustain good interface contact without serious cracks or delamination although material expansion or shrinkage would occur during the thermal cycle process. More importantly, it is noteworthy that the real temperature generally alters from 0 to 40^o^C when the devices are directly irradiated by sunlight [7,8]. Therefore, this durability during fluctuating temperature changes should be more important than the performance at low temperature in terms of near-space usage.

To better understand the temperature effect, we systematically investigated the device performance of the FOSCs at different temperatures. As shown in Fig. S7, *V*_OC_ gradually decreased as temperature increased from −60 to 40^o^C, while *J*_SC_ and FF slightly increased. The changing trend of *V*_OC_ could be described by variation of trap states, which could be described by the following equation [32]:


(1) \begin{eqnarray*} q{V}_{OC} = {E}_g - \frac{{\sigma _n^2 + \sigma _p^2}}{{2KT}} - {\rm{kTln}}\left( {\frac{{{N}_n{N}_p}}{{np}}} \right), \end{eqnarray*}

where *q* is the elementary charge, *E*_g_ is the energy gap, $\sigma $_n_$(\sigma $_p_) is the width of Gaussian density-of-state of acceptor and donor, *N_n_*(*N_p_*) is the effective states density of electron and hole, and *n*(*p*) is the free electron (hole) concentration. For the amorphous material-based device, *V*_OC_ was generally dependent on the value of $\sigma _n^2 + \sigma _p^2$. As temperature decreased, there would be more disordered tail states, resulting in larger $\sigma _n^2 + \sigma _p^2$, thereby *V*_OC_ decreased as the temperature rose. The decrease of *J*_SC_ and FF with the drop in temperature was observed, which might come from increased series resistance since the carrier mobility of the organic semiconductor materials would be lower as the temperature decreases. As a consequence, PCE increased with temperature increasing from −60 to 0^o^C and then decreased, with 0^o^C as a saturation. The temperature-dependent *V*_OC_, *J*_SC_, FF and PCE (Fig. 2e) clearly showed the relationship between the environmental temperature and device performance.

Light intensity-dependent *V*_OC_ and *J*_SC_ of the devices at 40, 0 and −60^o^C were then investigated (Figs S8 and S9) to understand the underlying reason for temperature-dependent performance. The increased slope of *V*_OC_*vs.* light intensity indicated that trap-assisted recombination became more dominant in the FOSCs at −60^o^C than at 0 and 40^o^C, which was similar to the results for the previous report [33]. Additionally, the electrochemical impedance spectroscopy (EIS) (Fig. S10, Table S2) showed larger recombination resistance at low temperatures. Based on the results of EIS and light-intensity *V*_OC_, we speculated large transfer resistance at −60^o^C from trap-assisted recombination was the main reason for low performance at low temperatures.

Similar temperature-dependent performance has been observed in silicon [34], CIGS [34] and perovskite solar cells [35], which has been ascribed to temperature-dependent carrier dynamics and bandgap change [36]. Recently, Tsoi *et al.* demonstrated that non-fullerene–acceptor-based OSCs have relatively lower performance at low temperatures (−100 to −20^o^C) than that at 0^o^C under the AM0 irradiation condition [33]. In addition, the result was highly dependent on the organic materials used [33], indicating material selection is critical for promoting low-temperature performance for future work.

The long-term stability of the flexible PBDB-T-2F : BTP-4F solar cells at different temperatures (−60°C, 25°C and 85°C) was investigated (Fig. 2f–h). As seen in this figure, we found both *V*_OC_ and FF were stable under continuous illumination at −60°C, whereas *J*_SC_ showed around 10% degradation, consequently leading to a 10% decline of efficiency after 300 h aging at −60°C. The slight degradation of *J*_SC_ might be attributed to the photochemical reaction between the metal oxide and the active layer during continuous illumination, which has been reported in our previous work [26]. In the case of 25^o^C, we found the device performance also declined by 10% under continuous illumination. At 85°C, the device showed a quick burn-in degradation process within 1 h followed by a long-time stable process. In addition, the recovery phenomenon of *J*_SC_ was observed during 85^o^C aging, which might be attributed to the morphology change of the organic photoactive layer [37]. Consequently, the device kept 80% of the initial performance after continuous illumination at 85°C for 300 h. These observations strongly demonstrated that FOSCs have reasonable long-term stability and continuous illumination at different temperatures, suggesting the excellent reliability of FOSCs during thermal cycling under near-space conditions [38]. Under comprehensive consideration of thermal cycle properties and UV resistance, the device structure of AgNWs/α-ZnO/ZnONP/C_60_/PBDB-T-2F:BTP-4F/C_60_/MoO_3_/Al was chosen for further *in situ* near-space measurement.

### *In situ* performance and stability test of FOSCs in stratospheric environment

To evaluate the device performance of FOSCs in a stratosphere environment, six PBDB-T-2F : BTP-4F FOSCs devices were launched at 35-km high altitude through a high-altitude balloon. Before flying, both sides of the FOSCs were encapsulated by water and oxygen barrier films, which resulted in a slight decrease in performance due to optical loss (Table S3).

**Figure 3.**
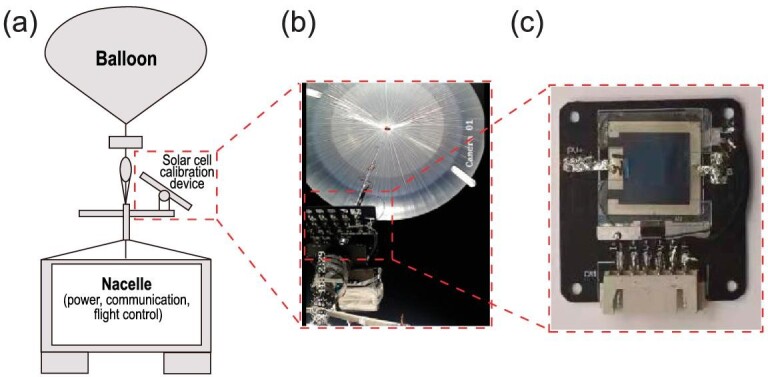
(a) Schematic diagram of the measurement instrument. (b) Photograph of the balloon for test at 35 km. (c) Photograph of the devices for test.

Figure 3a exhibits the schematic diagram of the high-altitude balloon measurement system. In detail, it consists of a high-altitude balloon, a cutter, a parachute, a measurement instrument, a pod, a control antenna, a buffered device and a cable. The high-altitude balloon is used to provide buoyancy to support the flight, the pod is used to store the electrical and communication units, and the cutter is used to stop the balloon flight at the end of the mission, or in an unexpected situation.

The cable is connected between the parachute and the pod, and consists of single or multiple rope belts (Fig. 3b). The high-altitude solar cell measurement is mainly composed of a support plate, a calibration plate, an azimuth stepper motor, a pitch stepper motor, a signal acquisition box, a solar tracking controller, a conductive slip ring and a cabin connector. The high-altitude *in situ* calibration instrument is clamped at the bottom of the connecting cable and begins to work after the balloon lifts off and reaches a certain flight height. The flight process is controlled by the sun tracking controller, which will track the position of the sun. The *J−V* testing system will collect the testing data and transmit them to the ground through the balloon communication link. In detail, each cell is separately connected with a separate *I−V* scan and signal sampling circuit to achieve an *I−V* curve within 1 s, and then the obtained *I−V* data are saved in the SD card and directly transmitted to the ground control room. Before the flight experiments, the solar cells were fixed on the circuit board (as shown in Fig. 3c). Here it is worth noting that although the balloon was continuously rotating in the sky due to wind, the use of a sun trajectory tracking system and photoelectronic tracking system could quickly calculate the accurate location and guide the balloon to quickly regulate its relative angle as long as the wind is not too strong. In this tracking system, a compass was used to detect the orientation and angle of the measurement instrument (as shown in Fig. S11), which would then guide the measurement instrument to locate the sun. On the other side, the incidence angle of the sun could be determined using the photoelectronic tracking system, which could correspondingly regulate the location to ensure that samples have been vertically illuminated.

**Figure 4.**
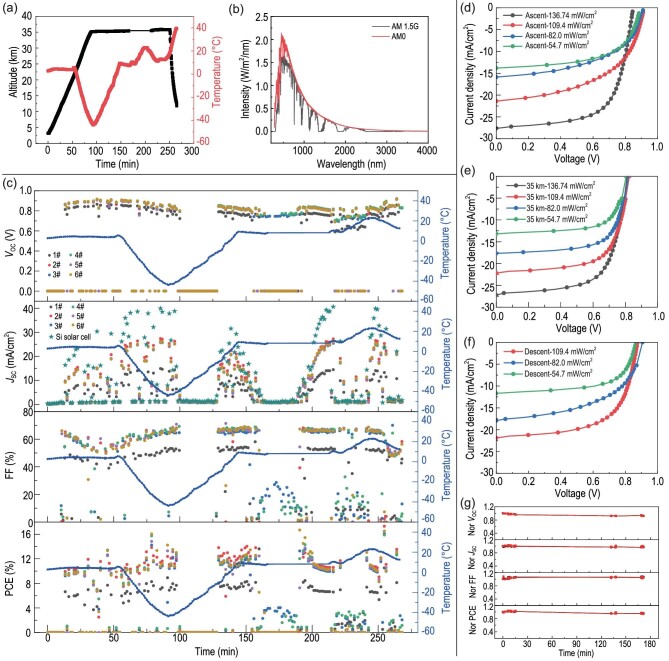
(a) Flying height and environment temperature. (b) The solar spectrum of AM0 and AM 1.5G. (c) Evolution of *V*_OC_, *J*_SC_, FF and PCE during the flight. (d–f) *J−V* curves of the FOSCs at different flying steps. (g) Device performance during 3 h of flight at 35 km.

Balloon altitude and environmental temperature were recorded by GPS and temperature sensors, respectively. As shown in Fig. 4a, the balloon rose to 35 km from 7 : 35 AM to 8 : 50 AM on 26 September 2021, and kept at 35-km altitude until 11 : 35 AM, and finally landed within 10 min. Regarding the temperature, we found it varied from 10°C to −40°C during the ascent step, varied from −8°C to 50°C during the level flight step, and then quickly decreased from 40°C to −40°C within 10 min, and finally rose to 10°C during the descent step. Solar irradiation intensity was estimated according to the current of the standard silicon solar cells. Figure 4b shows the solar spectrum of AM 1.5G and AM0. Overall, we found the AM0 solar spectrum contains much stronger UV irradiation than AM 1.5G spectrum. The standard irradiation intensity of AM 1.5G and AM0 is 100 and 136.7 mW/cm^2^, respectively. During the flight, the evolution of *V*_OC_, *J*_SC_, FF and PCE of the devices during temperature change was recorded and exhibited in Fig. 4c. However, we should point out that it is difficult to accurately analyse the impact of temperature on the device performance since the irradiation intensity changed due to the variation of incidence angle of sunlight, and the orientation and angle of the instrument. During the first 10 min, since the devices were far away from the sun at this step, both *V*_OC_ and *J*_SC_ were nearly 0. During the flying step from 10 to 100 min, the FOSCs gave a *V*_OC_ of about 0.80 V. During the level flying step, the devices showed a similar *V*_OC_ of 0.80 V. Regarding *J*_SC_, we found it gradually increased during the rising step, and varied largely from around 5.0 to 28.0 mA/cm^2^ during the whole process. Such a large variation of *J*_SC_ was caused by location changes during flight. FF was relatively stable during the whole process. In all, it was inspiring to find that the device showed an average performance of 11.0% to ∼13.0%, with the highest efficiency approaching 15%. Figure 4d–f shows the typica*l J−V* characteristics of the devices at the ascent, level flying, and descent steps with different irradiation intensities, and the corresponding performance parameters are listed in Table S4. In addition, the dependence of *J*_SC_ on incidence angle was investigated and exhibited in Fig. S12. We found the incidence angle changed from 4^o^ to 20^o^ during the flight, and correspondingly the *J*_SC_ of the FOSCs varied from 26.5 to 28.0 mA/cm^2^. Additionally, increased *J*_SC_ was observed when the incidence angle was smaller, and the maximum *J*_SC_ was achieved with an angle of 4^o^.

**Figure 5.**
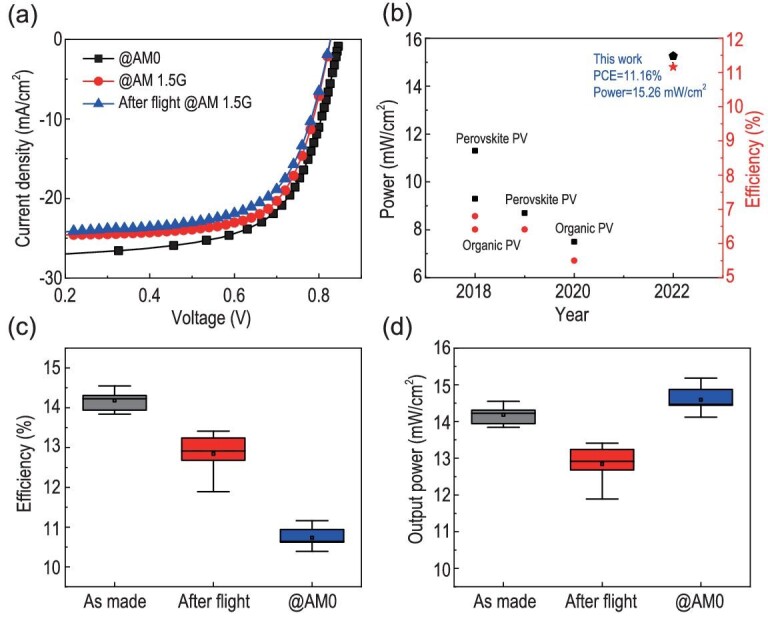
(a) *J−V* characteristics of the FOSCs in the terrestrial and near-space environments. (b) Summary of power and device efficiency of FOSCs in the stratospheric environment that have been reported [7–9]. (c) Device performance and (d) output power of the six individual FOSCs.

The performance evolution of the FOSCs during the 3-h flight at 35 km was also investigated and is shown in Fig. 4g. All the parameters, including *V*_OC_, *J*_SC_, FF and PCE presented a negligible decline, suggesting the FOSCs would be long-term stable in the stratospheric environment. The typical device performances of the six individual devices are listed in Table 1. We found these flexible devices showed similar performance, and the top device gave a *V*_OC_, *J*_SC_, FF and PCE of about 0.85 V, 26 mA/cm^2^, 65% and 11.16%, respectively. To evaluate the application potential of FOSCs in near-space, the device output power at AM0 and AM 1.5G was calculated and listed in Table 1. The typical *J−V* characteristics of FOSCs at AM0 and AM 1.5G are shown in Fig. 5a. As listed in Table 1, the FOSCs gave a higher output power under AM0 illumination than under AM 1.5G illumination. A highest power of 15.26 and 14.70 mW/cm^2^ was observed at AM0 and AM1.5G illumination, respectively. Such a high power and efficiency of FOSCs was the highest performance of OSCs in the stratospheric environment as far as we know [7,9], which was even higher than that of the perovskite solar cells (Table S5).

**Table 1.** Device performance of FOSCs at 35 km and in the lab.

Entry	*V*_OC_ (V)	*J*_SC_ (mA/cm^2^)	FF (%)	PCE (%) @AM0^a^	Power@AM0 (mW/cm^2^)	Power@AM1.5G (mW/cm^2^)
1	0.846	27.63	65.29	11.16	15.26	14.36
2	0.843	26.25	64.14	10.39	14.20	14.70
3	0.851	26.11	65.47	10.64	14.55	14.34
4	0.848	26.31	65.04	10.63	14.52	14.64
5	0.848	26.23	65.22	10.64	14.55	13.41
6	0.845	26.94	65.72	10.93	14.95	14.27

a AM0 spectrum: illumination intensity is 136.7 mW/cm^2^.

### Reliability checks of FOSCs after high-altitude test

After *in situ* performance measurement, we collected the devices and measured the performance under the illumination of AM 1.5G spectrum, and the performance is listed in Table S3. The performance and output power before and after flying and the performance in the terrestrial and near-space environments are shown in Fig. 5c and d. As shown in Fig. 5c, less than a 10% efficiency decline was observed after the stratospheric flight. Additionally, we found the degradation trend of these space-measured devices was similar to the control device (entry 7 in Table S3), suggesting natural degradation due to an inadequate water and oxygen barrier of encapsulation as the main reason for performance degradation. In other words, the FOSCs would be stable under near-space conditions if encapsulation is reliable. Based on the device performance of the fresh devices and the re-checked performance, we know the FOSCs could resist the extreme environment of near-space and keep stable.

## CONCLUSION

To evaluate the application potential of ultra-flexible OSCs in near space, both simulated experiments and *in situ* measurements at 35-km high altitude were investigated. The use of ZnO/C_60_ ETL was beneficial for enhancing UV durability. The terrestrial simulated experiments demonstrated that FOCSs with an inverted structure could withstand thermal cycles and UV irradiation. The flexible large-area OSCs gave an efficiency of higher than 11.0% and an outpower of higher than 15.0 mW/cm^2^ in the 35-km near-space environment, which corresponded to a power per weight of 3.32 kW/kg. In addition, the FOSCs kept stable during 3 h of flying at 35 km with only slight performance degradation. This work provided strong evidence of the application potential of large-area FOSCs with high performance and high power per weight as aerospace photovoltaics.

## Supplementary Material

nwac285_Supplemental_Files
