# Supplementary material for: In situ performance and stability tests of large-area flexible polymer solar cells in the 35-km stratospheric environment
Source: Natl Sci Rev. 2022 Dec 15;10(4):nwac285. doi: 10.1093/nsr/nwac285 (PMC10029844; doi:10.1093/nsr/nwac285)
Supplement: nwac285_Supplemental_Files [file nwac285_supplemental_files.zip › nwac285-Support Information-proof-SINANO.pdf]

Support Information

In situ performance and stability tests of large-area flexible polymer solar cells in the 35 km stratospheric environment

Zihan Xu[1,2], Guoning Xu[3,5,*], Qun Luo[1,2,*], Yunfei Han[1], Yu Tang[3], Ying Miao[3], Yongxiang Li[3], Jian Qin[1], Jingbo Guo[1], Wusong Zha[1], Chao Gong[1], Kun Lu[4], Jianqi Zhang[4], Zhixiang Wei[4,*], Rong Cai[3], Yanchu Yang[3], Zhaojie Li[3], and Chang-Qi Ma[1,2,*]

[1]i-Lab & Printable Electronic Center, Suzhou Institute of Nano-Tech and Nano-Bionics, Chinese Academy of Sciences, Suzhou 215123, China;

[2]School of Nano-Tech and Nano-Bionics, University of Science and Technology of China, Hefei 230027, China;

[3]Aerospace Information Research Institute, Chinese Academy of Sciences, Beijing 100094, China;

[4]CAS Key Laboratory of Nanosystem and Hierarchical Fabrication, National Center for Nanoscience and Technology, Beijing 100190, China;

[5] University of Chinese Academy of Sciences, Beijing 100049, China

***Corresponding authors**. E-mails: xugn@aircas.ac.cn; qluo2011@sinano.ac.cn; weizx@nanoctr.cn; cqma2011@sinano.ac.cn

Materials Processing and Device Fabrication: Silver nanowires dispersed in deionized water and diluted with isopropanol (IPA) were purchased from H&C Advanced Materials Ltd (Nanchang, China). The zinc acetate was supplied by Aladdin Ltd. The 2-methoxyethanol was purchased from J&K Scientific. Ethanol amine was purchased from Sigma-Aldrich. The donor Poly[[4,8-bis[5-(2-ethylhexyl)-4-fluoro-2-thienyl]benzo[1,2-*b*:4,5-*b*']dithiophene-2,6-diyl]-2,5-thiophenediyl[5,7-bis(2-ethylhexyl)-4,8-dioxo-4H,8H-benzo[1,2-c:4,5-c']dithiophene-1,3-diyl]-2,5-thiophenediyl] (PBDB-T-2F), acceptor (2,20-((2Z,20Z)-((12,13-bis(2-ethylhexyl)-3,9-diundecyl-12,13-dihydro-[1,2,5]thiadiazolo[3,4-e]thieno[2,"30':4',50] thieno[20,30:4,5]pyrrolo[3,2-*g*]thieno[20,30:4,5]thieno[3,2-*b*]indole-2,10-diyl)bis(methanylylidene))bis(5,6-difluoro-3-oxo-2,3-dihydro-1H-indene-2,1-diylidene))dimalononitrile) (BTP-4F) were purchased from Solarmer Material Inc., Beijing. Chloroform (CF) were purchased from Yonghua Chemical Co. Ltd. 1,8-Diiodooctane (DIO) and 1-chloronaphthalene (CN) were purchased from Sigma-Aldrich. UV curable glue was purchased from NORLAND (Norland optical Adhesive 61).

PET/AgNWs electrodes were fabricated by gravure printing through the same route as reported in our previous work[1]. The AgNWs were treated by UV ozone for 5 minutes. Then, an amorphous ZnO (α-ZnO) modification layer was deposited on the top of AgNWs through spin-coating with 0.1M ZnO precursor at 2000 rpm/min for 45 s, and followed by annealing at 150 °C for 30 min[2]. Next, ZnO nanoparticles (named ZnO NP, dispersed in CH_3OH, 15 mg/mL) were spin-coated on the α-ZnO films at 2000

rpm/min for 45 s, and the films were annealed at 130 °C for 10 min. Subsequently, films were transferred into a glove box filled with nitrogen to fabricate the active layer. The PBDB-T-2F: BTP-4F solution (1:1.2, w/w, 17.6 mg/mL in CF with 0.5% CN) was spin-coated on the ZnO NPs films to achieve the organic photoactive layer. The active layers were annealed at 100 ºC for 10 min. Finally, 5 nm C_{60}, 20 nm MoO_3 and 150 nm Al were deposited on the active layer through vacuum evaporation (~4×10^{-4} Pa). The effective area of the flexible organic solar cells is 0.64 cm^2. For the device encapsulation, the barrier films with water oxygen permeability of ~10^{-5} g /cm^2/day were pasted on both sides of the flexible devices through UV glue. The UV glue was cross-linked after irradiation under an UV lamp with wavelength of 365 nm for 5 min.

Characterization: The current density-voltage (*J-V*) measurements were tested in a N_2-filled glove box with Keithley 2400 source meter under AM 1.5G illumination (100 mW/cm^2, Zolix SS150). The long-term stability of the encapsulated devices under different temperatures was recorded through period *J-V* sweeps under continuous illumination with devices stored in a LED light source integrated climate chamber (D&R PVLT-9001-16A, Co. Ltd., Suzhou). The *J-V* characteristics were measured through *I-V* sweeps each 5-6 min, during which an external load that fits the maximum power point (MPP) of each cell was attached to the cell to ensure the cell is under operation at MPP point. The current density-voltage (*J-V*) measurements at 35 km were measured as follow. In detail, the FOSCs were welded on the circuit board, and the circuit board was connected with a data acquisition instrument. The high-precision test system of solar cells on the platform collects the data of the calibrated cells and

transferred data to the ground in real-time through the balloon communication link.

Table S1. Device performance of the PBDB-T-2F: BTP-4F FOSCs that measured under AM 1.5G condition.

**Substrate Thickness (μm)[a]**	V_{OC} **(V)**	J_{SC} **(mA/cm²)**	**FF (%)**	**PCE[b] (%)**	PCE^{c}_{max} **(%)**
125	0.820±0.001	24.81±0.48	69.77±0.80	14.19±0.25	14.61
38	0.825±0.001	24.51±0.36	72.97±0.73	14.76±0.24	15.01

[a] Substrate thickness; [b] Averaged performance over 10 individual cells; [c] PCE of the best cell. The device area is 0.64 cm^2

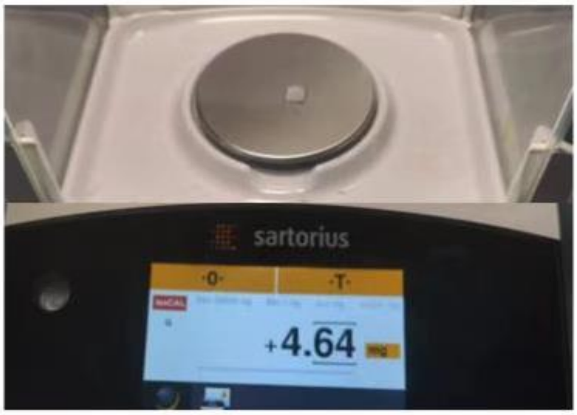

Figure S1 Photographs of the FOSCs with 38 μm-thick PET substrate.

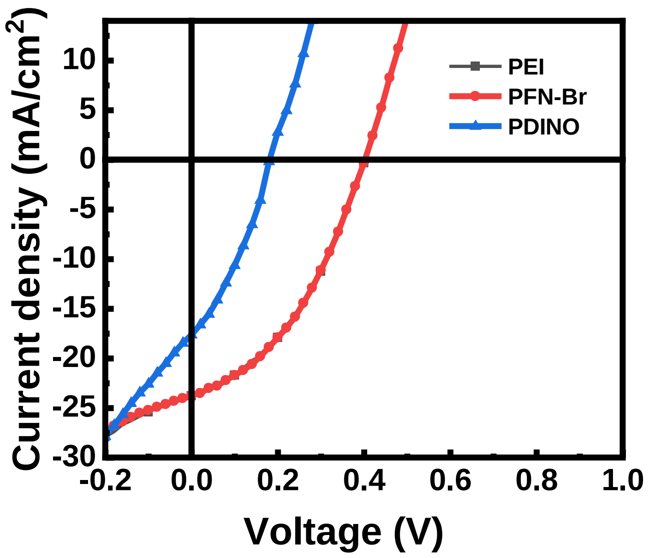

Figure S2 *J-V* characteristics of the flexible OSCs with different ETLs.

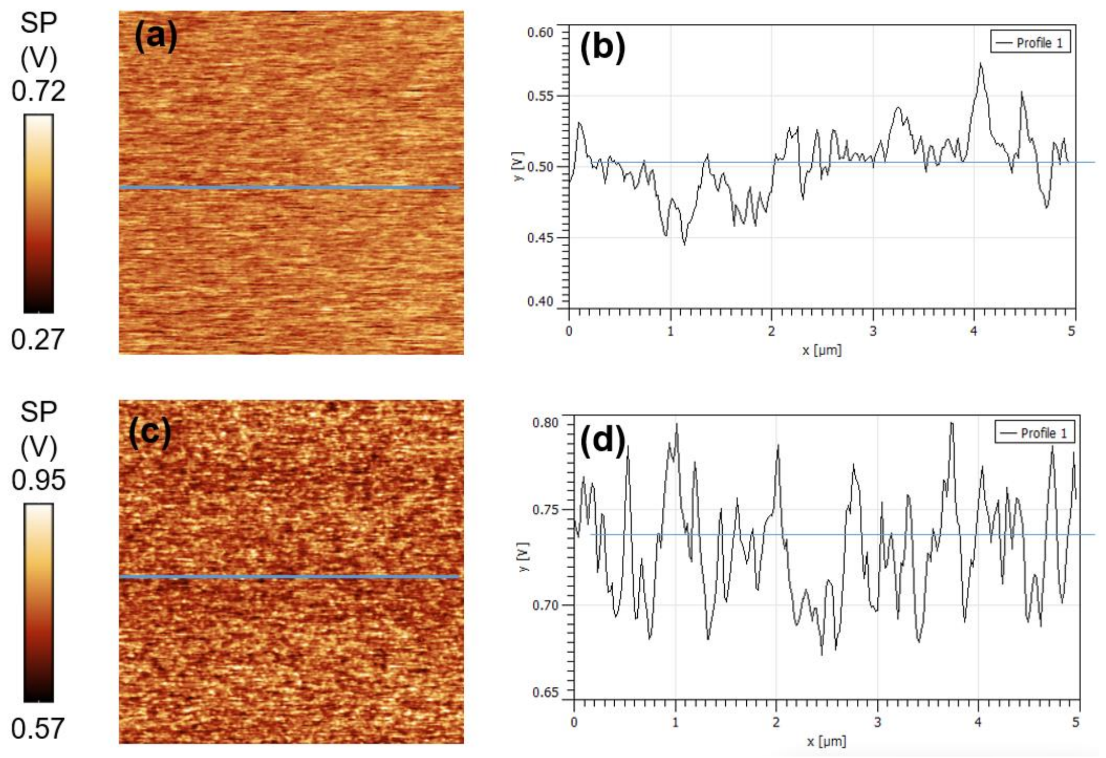

Figure S3 Surface potential of the pristine (a,b) ZnO and (c,d) ZnO after UV soaking for 30 h.

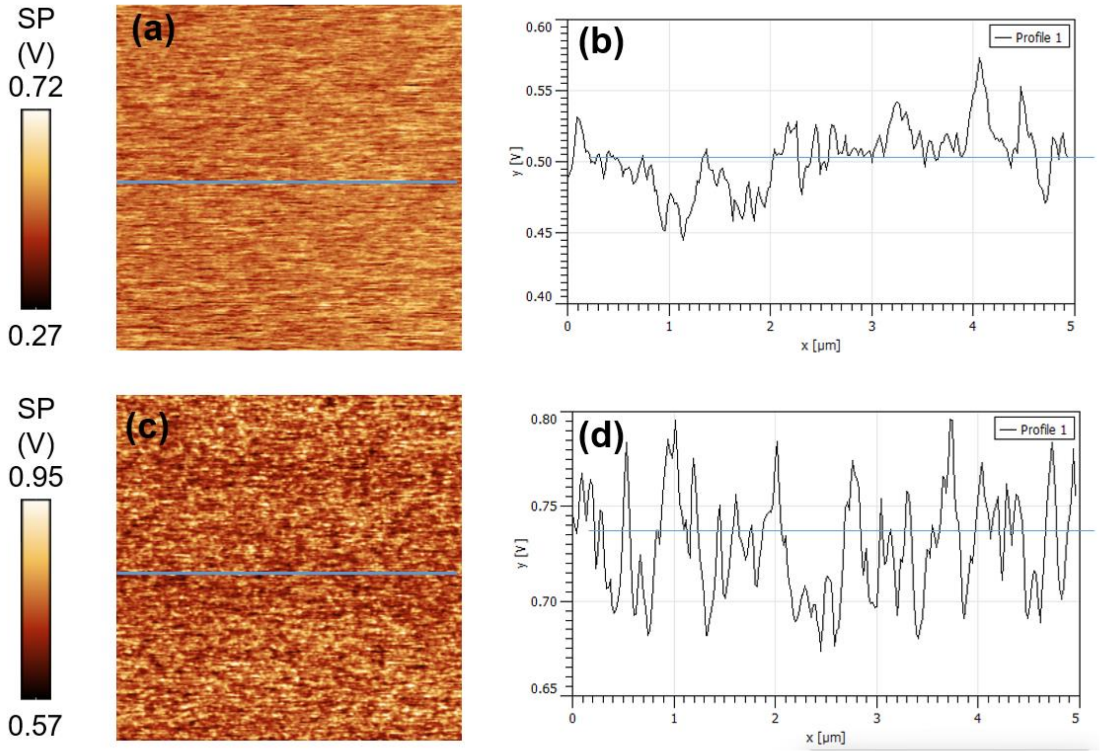

Figure S4 Surface potential of the pristine (a,b) ZnO/C_{60} and (c,d) after UV soaking for 30 h.

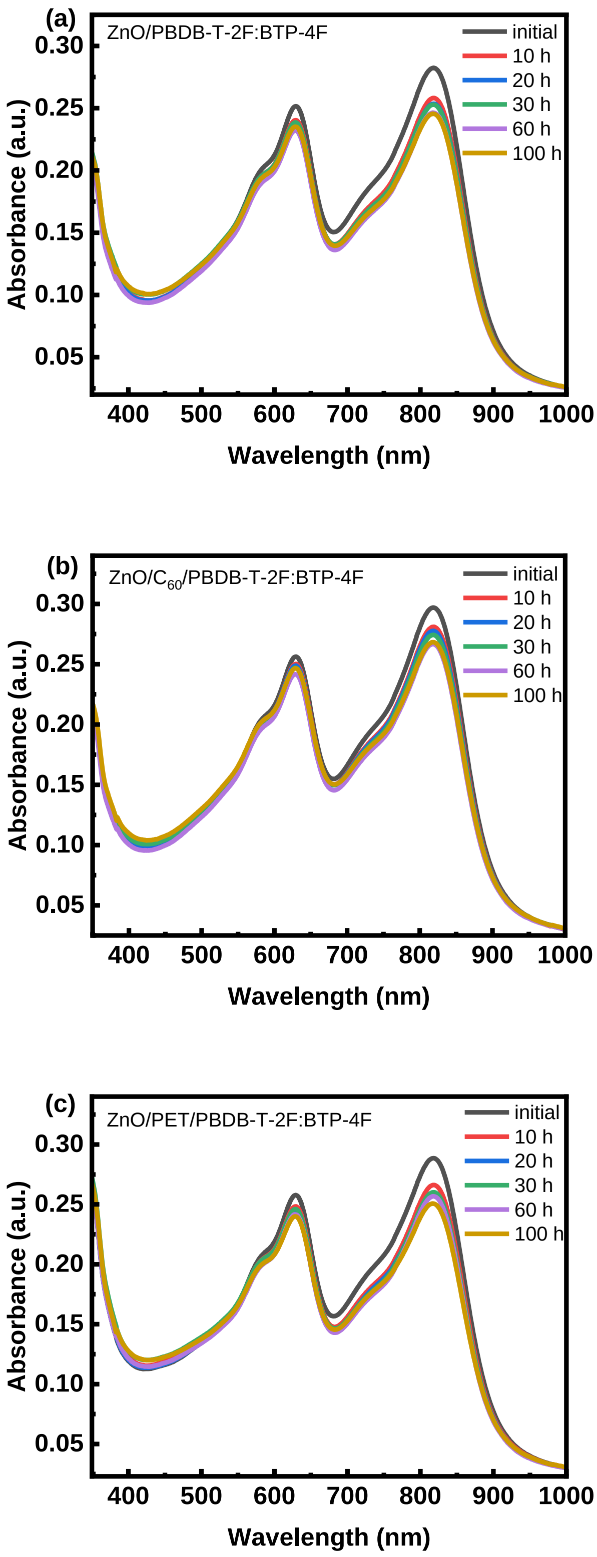

Figure S5 UV-vis absorbance of the (a) ZnO/PBDB-T-2F:BTP-4F, (b) ZnO/C_{60}/PBDB-T-2F:BTP-4F, and (c) ZnO/PET/PBDB-T-2F:BTP-4F during UV irridation.

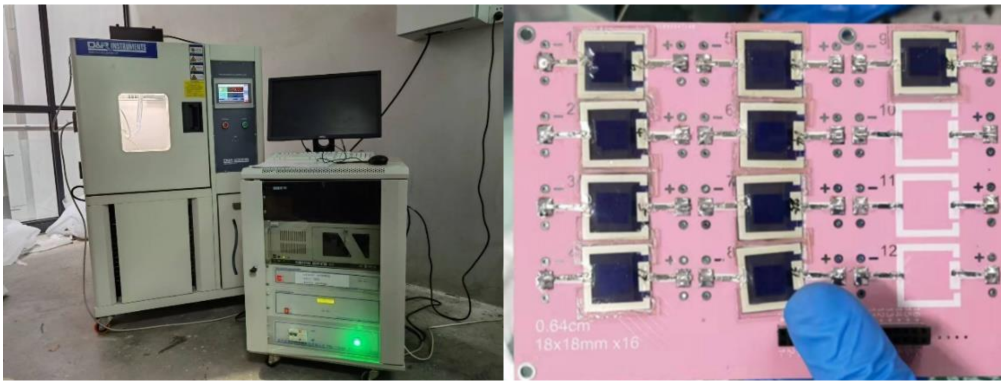

Figure S6 Photographs of the *in situ J*-V curves measurements instrument during thermal cycling, and the devices.

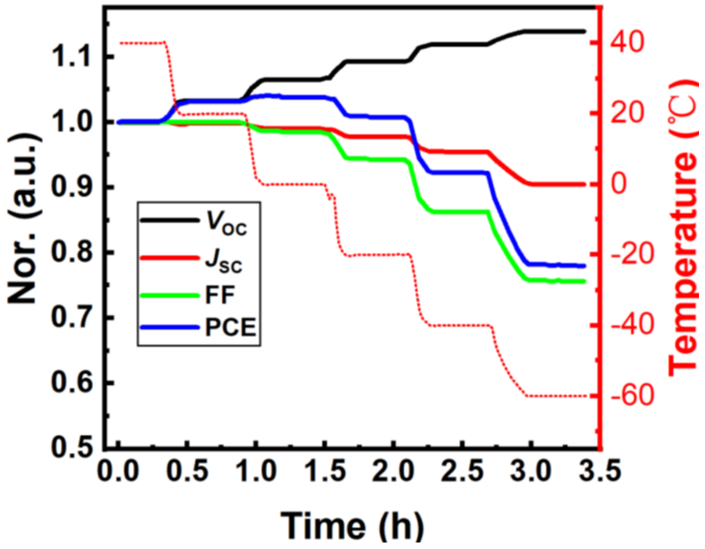

Figure S7 Evolution of performance parameter at different temperatures (the performance was normalized at the value of 40 °C).

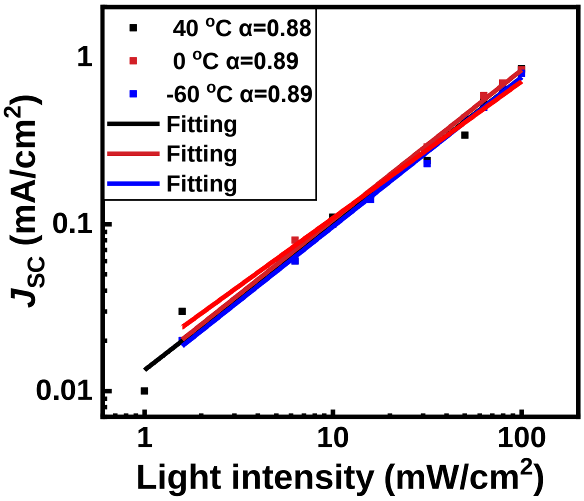

Figure S8 Light-intensity-dependent J_{SC} at different temperatures.

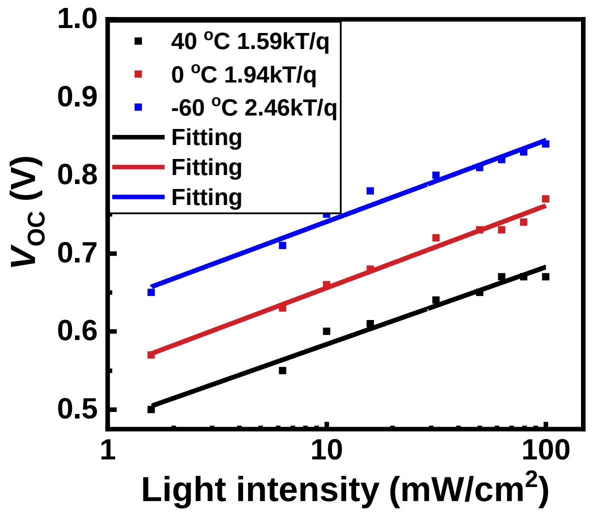

Figure S9 Light-intensity-dependent V_{OC} at different temperatures.

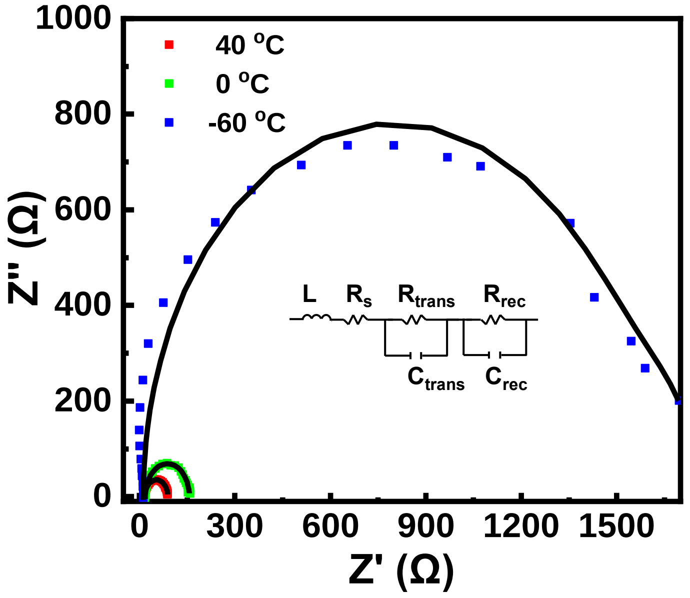

Figure S10 The electrochemical impedance spectroscopy (EIS) of the FOSCs at different temperatures.

Table S2. Fitting results of the electrochemical impedance spectroscopy of the FOSCs at different temperatures.

Temperature (°C)	R_s (Ω)	R_{trans} (Ω)	R_{rec} (Ω)	C_{trans} (F)	C_{rec} (F)	L (H)
40	15.88	16.85	56.65	5.99×10^{7}	1.12×10^{7}	0.6
0	17.68	139.4	87.28	5.77×10^{8}	1.12×10^{4}	1.78
-60	11.93	1507	239.6	1.72×10^{8}	9.52×10^{7}	6.99

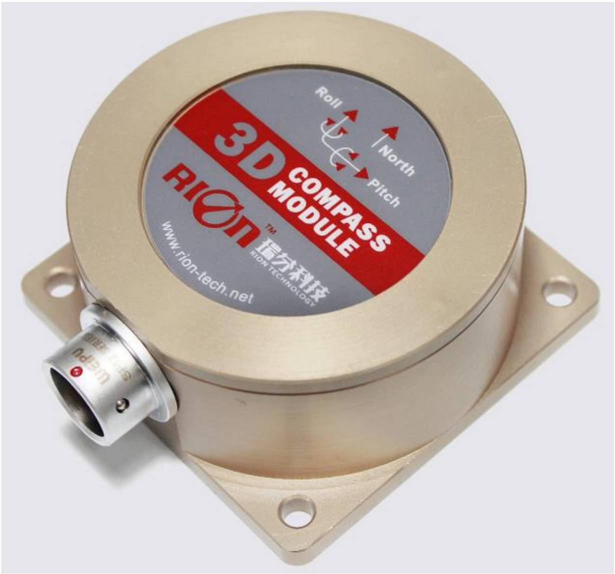

Figure S11 The photographs of the compass that was used to track the orientation of the measurement instrument.

Table S3. Device performance of flexible PBDB-T-2F:BTP-4F solar cells as fabricated and after encapsulation and welding.

Entry	Process	V_{OC} (V)	J_{SC} (mA/cm^2)	FF (%)	$PCE_{AM1.5G}$ (%)
1	As fabricated	0.828	24.56	70.64	14.36
	After encapsulation	0.824	24.41	70.50	14.19
	After flight	0.826	23.85	66.66	13.12
2	As fabricated	0.831	25.12	70.45	14.70
	After encapsulation	0.827	25.09	70.19	14.55
	After flight	0.822	24.52	65.69	13.24
3	As fabricated	0.831	24.53	70.39	14.34
	After encapsulation	0.826	24.47	70.50	14.26
	After flight	0.815	23.23	62.78	11.89
4	As fabricated	0.828	25.22	70.09	14.64
	After encapsulation	0.826	24.84	69.74	14.31
	After flight	0.827	24.59	65.87	13.41
5	As fabricated	0.830	24.61	69.92	14.27
	After encapsulation	0.825	24.32	70.36	14.12
	After flight	0.817	23.51	66.04	12.68
6	As fabricated	0.827	24.45	70.01	14.16
	After encapsulation	0.827	24.07	69.99	13.94
	After flight	0.820	24.62	0.654	13.19
7[a]	As fabricated	0.828	24.67	70.09	14.28
	After encapsulation	0.825	24.25	69.12	13.84
	After flight	0.804	24.06	66.37	12.71

[a]: Control device that stored in air for one week.

Table S4. Device performance at different stages.

Stage	Irradiation intensity (mW/cm²)	V_{OC} (V)	J_{SC} (mA/cm²)	FF (%)	PCE (%)
**Ascent**	136.7	0.846	27.63	64.14	10.50
	109.4	0.911	21.39	52.83	8.93
	82.0	0.911	15.89	51.71	8.33
	54.7	0.886	13.81	59.24	11.84
**Level flying**	136.7	0.809	27.16	64.58	9.89
	109.4	0.826	21.81	65.52	10.41
	82.0	0.824	17.84	65.83	10.81
	54.7	0.820	13.06	66.51	11.41
**Descent**	109.4	0.875	21.91	61.37	9.91
	82.0	0.907	17.91	51.76	10.07
	54.7	0.867	11.69	61.18	11.13

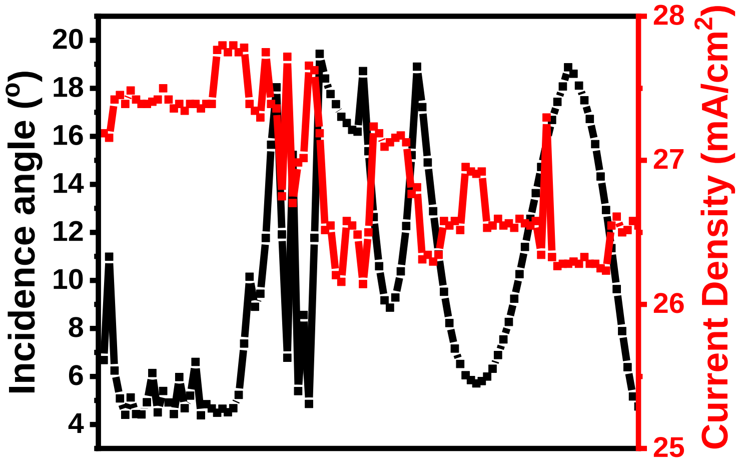

Figure S12 Incidence angle of the sunlight during the in-situ experiment and the variation of J_{SC}.

Table S5. Summary of the device performance and power of the organic solar cells and perovskite solar cells in the stratospheric environment and far-space environment.

Year	Device type	Device area (cm^2)	Efficiency (%)	Power (mW/cm^2)	Ref
2018	$MAPbI_3$ (rigid)	N/A	6.41	11.30	[3]
2019	$FA_{0.9}Cs_{0.1}PbI_3$	1.00	6.41	8.70	[4]
2020	PTB7-Th:$PC_{71}BM$	N/A	5.50	7.50	[5]
2020	Perovskite	N/A	10.20	14.00	[5]
2022	PDBD-T-2F:BTP-4F (Flexible, this work)	0.64	11.16	15.26	This work

References:

[1] Wang Z, Han Y, Yan L, *et al.* High-power conversion efficiency of 13.61% for 1 cm^2 flexible polymer solar cells based on patternable and mass-producible gravure-printed silver nanowire electrodes. *Adv Funct Mater* 2020; 31: 2007276.

[2] Pan W, Han Y, Wang Z, *et al.* An efficiency of 14.29% and 13.08% for 1 cm^2 and 4 cm^2 flexible organic solar cells enabled by sol–gel ZnO and ZnO nanoparticle bilayer electron transporting layers. *J Mater Chem A* 2021; 9: 16889-16897.

[3] Cardinaletti I, Vangerven T, and Nagels S*, et al.* Organic and perovskite solar cells for space applications. *Sol Energy Mater Sol Cells* 2018; 182: 121-7.

[4] Tu Y, Xu G, and Yang X*, et al.* Mixed-cation perovskite solar cells in space. *Sci China Phys Mech* 2019; 62(7) 974221.

[5] Reb LK, Böhmer M, and Predeschly B*, et al.* Perovskite and organic solar cells on a rocket flight. *Joule* 2020; 4: 1880-92.
