# Supplementary material for: In situ performance and stability tests of large-area flexible polymer solar cells in the 35-km stratospheric environment
Source: Natl Sci Rev. 2022 Dec 15;10(4):nwac285. doi: 10.1093/nsr/nwac285 (PMC10029844; doi:10.1093/nsr/nwac285)
Supplement: nwac285_Supplemental_Files [file nwac285_supplemental_files.zip › Supplementary data.docx]

**Support Information**

***In situ* performance and stability tests of large-area flexible polymer solar cells in the 35 km stratospheric environment**

Zihan Xu^1,2^, Guoning Xu^3,5,*^, Qun Luo^1,2,*^, Yunfei Han^1^, Yu Tang^3^, Ying Miao^3^, Yongxiang Li^3^, Jian Qin^1^, Jingbo Guo^1^, Wusong Zha^1^, Chao Gong^1^, Kun Lu^4^, Jianqi Zhang^4^, Zhixiang Wei^4,*^, Rong Cai^3^, Yanchu Yang^3^, Zhaojie Li^3^, and Chang-Qi Ma^1,2,*^

^1^i-Lab & Printable Electronic Center, Suzhou Institute of Nano-Tech and Nano-Bionics, Chinese Academy of Sciences, Suzhou 215123, China;

^2^School of Nano-Tech and Nano-Bionics, University of Science and Technology of China, Hefei 230027, China;

^3^Aerospace Information Research Institute, Chinese Academy of Sciences, Beijing 100094, China;

^4^CAS Key Laboratory of Nanosystem and Hierarchical Fabrication, National Center for Nanoscience and Technology, Beijing 100190, China;

^5^ University of Chinese Academy of Sciences, Beijing 100049, China

***Corresponding authors**. E-mails: [xugn@aircas.ac.cn](mailto:xugn@aircas.ac.cn); [qluo2011@sinano.ac.cn](mailto:qluo2011@sinano.ac.cn); [weizx@nanoctr.cn](mailto:weizx@nanoctr.cn); [cqma2011@sinano.ac.cn](mailto:cqma2011@sinano.ac.cn)

**Materials Processing and Device Fabrication:** Silver nanowires dispersed in deionized water and diluted with isopropanol (IPA) were purchased from H&C Adv. Mater. Co. Ltd (Nanchang, China). The zinc acetate was supplied by Aladdin Ltd. The 2-methoxyethanol was purchased from J&K Scientific. Ethanol amine was purchased from Sigma-Aldrich. The donor Poly[[4,8-bis[5-(2-ethylhexyl)-4-fluoro-2-thienyl]­benzo[1,2-*b*:4,5-*b*']dithiophene-2,6-diyl]-2,5-thiophenediyl[5,7-bis(2-ethylhexyl)-4,8-dioxo-4H,8H-benzo[1,2-c:4,5-*c*']dithiophene-1,3-diyl]-2,5-thiophenediyl] (PBDB-T-2F ), acceptor (2,20-((2Z,20Z)-((12,13-bis(2-ethylhexyl)-3,9-diundecyl-12,13-dihydro-[1,2,5]thiadiazolo[3,4-e]thieno[2,''30':4',50] thieno[20,30:4,5]pyrrolo[3,2-*g*]thieno[20,30:4,5]thieno[3,2-*b*]indole-2,10-diyl)bis(methanylylidene))bis(5,6-difluoro-3-oxo-2,3-dihydro-1H-indene-2,1-diylidene))dimalononitrile) (BTP-4F) were purchased from Solarmer Material Inc., Beijing. Chloroform (CF) were purchased from Yonghua Chemical Co. Ltd*.* 1,8-Diiodooctane (DIO) and 1-chloronaphthalene (CN) were purchased from Sigma-Aldrich. UV curable glue were purchased from NORLAND (Norland Optical Adhesive 61).

PET/AgNWs electrodes were fabricated by gravure printing through the same route as reported in our previous work[1]. The AgNWs were treated by UV ozone for 5 minutes. Then, an amorphous ZnO (α-ZnO) modification layer was deposited on the top of AgNWs through spin-coating with 0.1M ZnO precursor at 2000 rpm/min for 45 s, and followed by annealing at 150 ℃ for 30 min[2]. Next, ZnO nanoparticles (named ZnO NP, dispersed in CH_3_OH, 15 mg/mL) were spin-coated on the α-ZnO films at 2000 rpm/min for 45 s, and the films were annealed at 130 ℃ for 10 min. Subsequently, films were transferred into a glove box filled with nitrogen to fabricate the active layer. The PBDB-T-2F: BTP-4F solution (1:1.2, w/w, 17.6 mg/mL in CF with 0.5% CN) was spin-coated on the ZnO NPs films to achieve the organic photoactive layer. The active layers were annealed at 100 ⁰C for 10 min. Finally, 20 nm MoO_3_ and 150 nm Al were deposited on the active layer through vacuum evaporation (~4×10^-4^ Pa). The effective area of the flexible organic solar cells is 0.64 cm^2^. For the device encapsulation, the barrier films with water oxygen permeability of ~10^-5^ g /cm^2^/day were pasted on both sides of the flexible devices through UV glue. The UV glue was cross-linked after irradiation under an UV lamp with wavelength of 365 nm for 5 min.

**Characterization:** The current density-voltage (*J-V*) measurements were tested in a N_2_-filled glove box with Keithley 2400 source meter under AM 1.5G illumination (100 mW/cm^2^, Zolix SS150). The long-term stability of the encapsulated devices under different temperatures was recorded through period *J-V* sweeps under continuous illumination with devices stored in a LED light source integrated climate chamber (D&R PVLT-9001-16A, Co. Ltd., Suzhou). The *J*-*V* characteristics were measured through *I-V* sweeps each 5-6 min, during which an external load that fits the maximum power point (MPP) of each cell was attached to the cell to ensure the cell is under operation at MPP point. The current density-voltage (*J-V*) measurements at 35 km were measured as follow. In detail, the FOSCs were welded on the circuit board, and the circuit board was connected with a data acquisition instrument. The high-precision test system of solar cells on the platform collects the data of the calibrated cells and transferred data to the ground in real-time through the balloon communication link.

**Table S1.** Device performance of the PBDB-T-2F: BTP-4F FOSCs that measured under AM 1.5G condition.

| **Substrate Thickness (μm)^a^** | ***V*_OC_**  **(V)** | ***J*_SC_**  **(mA/cm^2^)** | **FF**  **(%)** | **PCE***^b^*  **(%)** | **PCE^c^_max_**  **(%)** |
| --- | --- | --- | --- | --- | --- |
| 125 | 0.820±0.001 | 24.81±0.48 | 69.77±0.80 | 14.19±0.25 | 14.61 |
| 38 | 0.825±0.001 | 24.51±0.36 | 72.97±0.73 | 14.76±0.24 | 15.01 |

^a^ Substrate thickness; ^b^ Averaged performance over 10 individual cells; ^c^ PCE of the best cell. The device area is 0.64 cm^2^

**Table S2.** Fitting results of the electrochemical impedance spectroscopy of the FOSCs at different temperatures.

| Temperature  (^o^C) | R_s_ (Ω) | R_trans_  **(**Ω**)** | R_rec_  **(**Ω**)** | C_trans_  (F) | C_rec_  (F) | L  (H) |
| --- | --- | --- | --- | --- | --- | --- |
| 40 | 15.88 | 16.85 | 56.65 | 5.99× 10^7^ | 1.12× 10^7^ | 0.6 |
| 0 | 17.68 | 139.4 | 87.28 | 5.77× 10^8^ | 1.12× 10^4^ | 1.78 |
| -60 | 11.93 | 1507 | 239.6 | 1.72×10^8^ | 9.52 ×10^7^ | 6.99 |

**Table S3**. Device performance of flexible PBDB-T-2F:BTP-4F solar cells as fabricated and after encapsulation and welding.

| **Entry** | **Process** | ***V*_OC_**  **(V)** | ***J*_SC_**  **(mA/cm^2^)** | **FF**  **(%)** | **PCE_AM1.5G_**  **(%)** |
| --- | --- | --- | --- | --- | --- |
| 1 | As fabricated | 0.828 | 24.56 | 70.64 | 14.36 |
|  | After encapsulation | 0.824 | 24.41 | 70.50 | 14.19 |
|  | After flight | 0.826 | 23.85 | 66.66 | 13.12 |
| 2 | As fabricated | 0.831 | 25.12 | 70.45 | 14.70 |
|  | After encapsulation | 0.827 | 25.09 | 70.19 | 14.55 |
|  | After flight | 0.822 | 24.52 | 65.69 | 13.24 |
| 3 | As fabricated | 0.831 | 24.53 | 70.39 | 14.34 |
|  | After encapsulation | 0.826 | 24.47 | 70.50 | 14.26 |
|  | After flight | 0.815 | 23.23 | 62.78 | 11.89 |
| 4 | As fabricated | 0.828 | 25.22 | 70.09 | 14.64 |
|  | After encapsulation | 0.826 | 24.84 | 69.74 | 14.31 |
|  | After flight | 0.827 | 24.59 | 65.87 | 13.41 |
| 5 | As fabricated | 0.830 | 24.61 | 69.92 | 14.27 |
|  | After encapsulation | 0.825 | 24.32 | 70.36 | 14.12 |
|  | After flight | 0.817 | 23.51 | 66.04 | 12.68 |
| 6 | As fabricated | 0.827 | 24.45 | 70.01 | 14.16 |
|  | After encapsulation | 0.827 | 24.07 | 69.99 | 13.94 |
|  | After flight | 0.820 | 24.62 | 0.654 | 13.19 |
|  | As fabricated | 0.828 | 24.67 | 70.09 | 14.28 |
| 7^a^ | After encapsulation | 0.825 | 24.25 | 69.12 | 13.84 |
|  | After flight | 0.804 | 24.06 | 66.37 | 12.71 |

^a:^ Control device that stored in air for one week.

**Table S4.** Device performance at different stages.

| **Stage** | **Irradiation intensity**  **(mW/cm^2^)** | ***V*_OC_**  **(V)** | ***J*_SC_**  **(mA/cm^2^)** | **FF**  **(%)** | **PCE**  **(%)** |
| --- | --- | --- | --- | --- | --- |
| **Ascent** | 136.7 | 0.846 | 27.63 | 64.14 | 10.50 |
|  | 109.4 | 0.911 | 21.39 | 52.83 | 8.93 |
|  | 82.0 | 0.911 | 15.89 | 51.71 | 8.33 |
|  | 54.7 | 0.886 | 13.81 | 59.24 | 11.84 |
| **Level**  **flying** | 136.7 | 0.809 | 27.16 | 64.58 | 9.89 |
|  | 109.4 | 0.826 | 21.81 | 65.52 | 10.41 |
|  | 82.0 | 0.824 | 17.84 | 65.83 | 10.81 |
|  | 54.7 | 0.820 | 13.06 | 66.51 | 11.41 |
| **Descent** | 109.4 | 0.875 | 21.91 | 61.37 | 9.91 |
|  | 82.0 | 0.907 | 17.91 | 51.76 | 10.07 |
|  | 54.7 | 0.867 | 11.69 | 61.18 | 11.13 |

**Table S5**. Summary of the device performance and power of the organic solar cells and perovskite solar cells at stratospheric environment and far-space environment.

| **Year** | **Device type** | **Device area**  **(cm^2^)** | **Efficiency (%)** | **Power**  **(mW/cm^2^)** | **Ref** |
| --- | --- | --- | --- | --- | --- |
| 2018 | MAPbI_3_ (rigid) | N/A | 6.41 | 11.30 | [3] |
| 2019 | FA_0.9_Cs_0.1_PbI_3_ | 1.00 | 6.41 | 8.70 | [4] |
| 2020 | PTB7-Th:PC_71_BM | N/A | 5.50 | 7.50 | [5] |
| 2020 | Perovskite | N/A | 10.20 | 14.00 | [5] |
| 2022 | PDBD-T-2F:BTP-4F  (Flexible, this work) | 0.64 | 11.16 | 15.26 | This work |


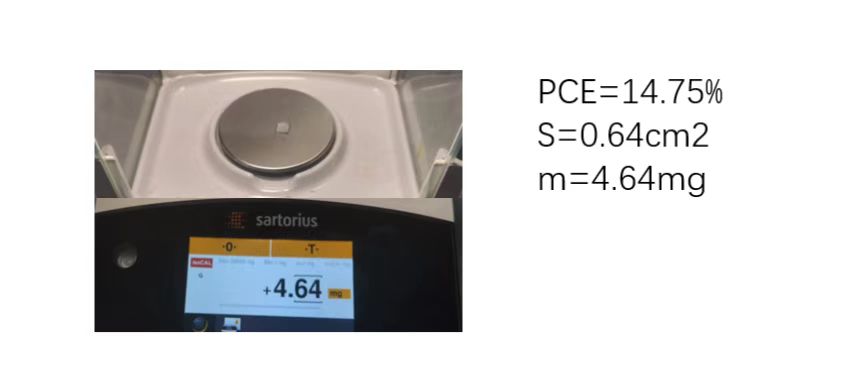


**Figure S1** Photographs of the FOSCs with 38 μm-thick PET substrate.

**Figure S2** *J-V* characteristics of the flexible OSCs with difffent ETLs.


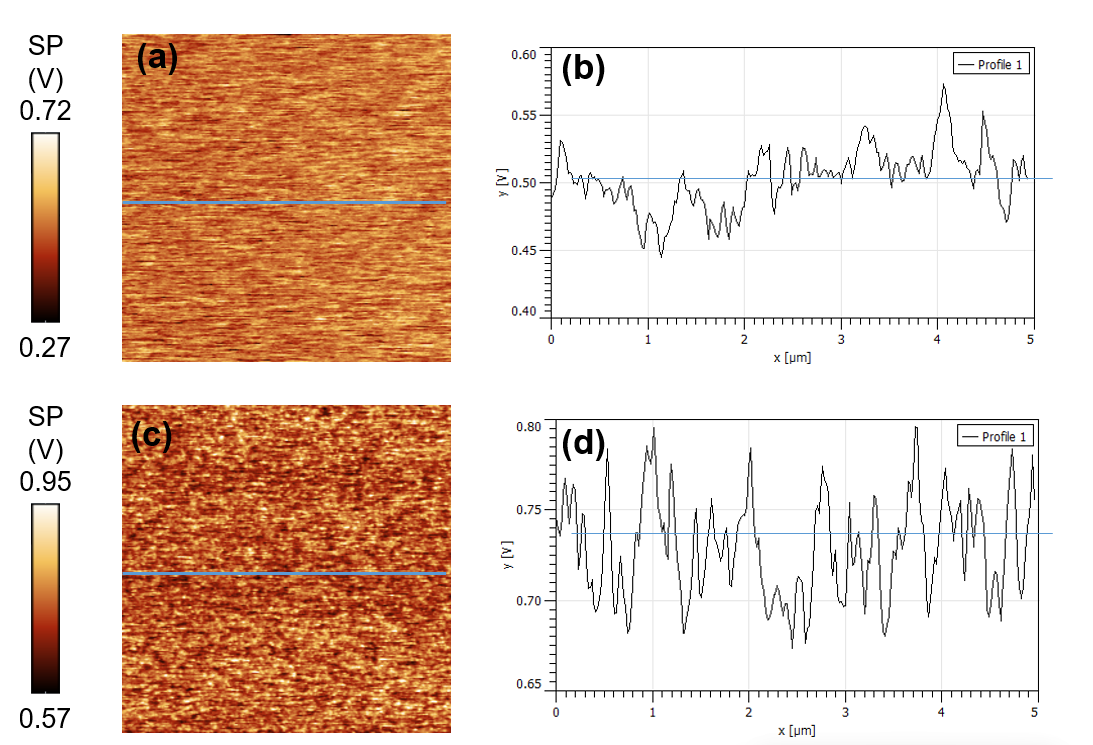


**Figure S3** Surface potential of the pristine (a,b) ZnO and (c,d) ZnO after UV soaking for 30 h.


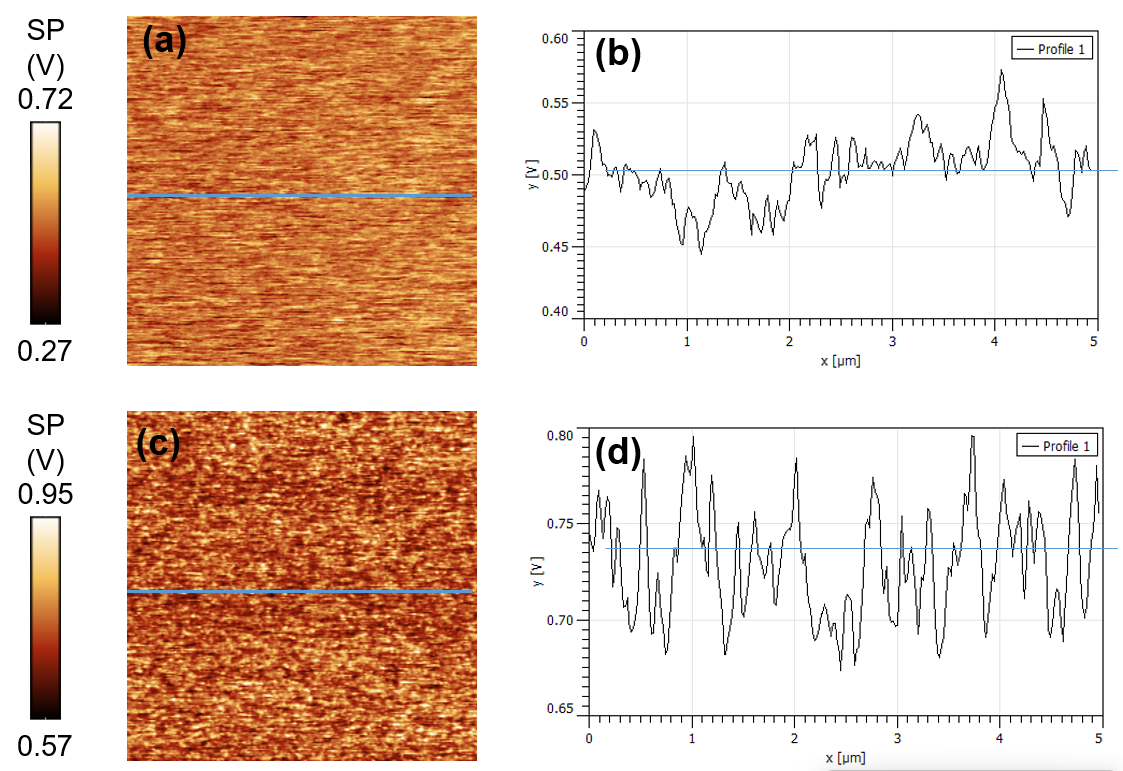


**Figure S4** Surface potential of the pristine (a,b) ZnO/C_60_ and (c,d) after UV soaking for 30 h.

**Figure S5** UV-vis absorbance of the (a) ZnO/PBDB-T-2F:BTP-4F, (b) ZnO/C_60_/PBDB-T-2F:BTP-4F, and (c) ZnO/PET/PBDB-T-2F:BTP-4F during UV irridation.


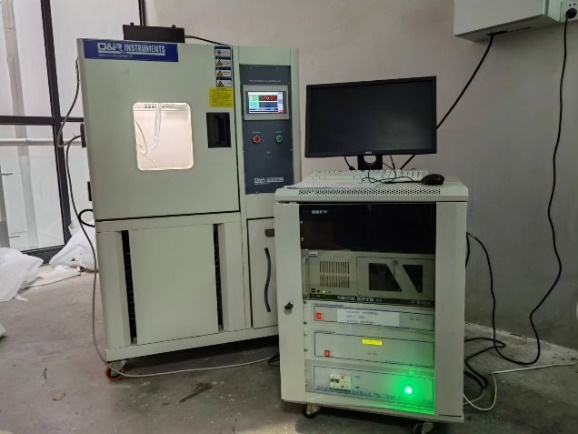

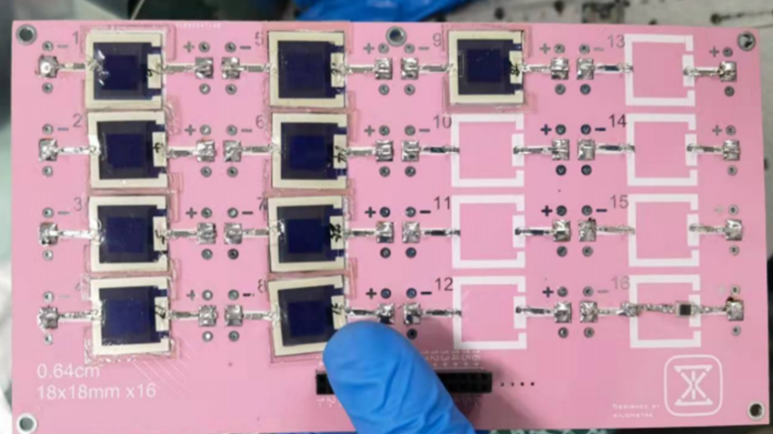


**Figure S6** Photogrpahs of the *in situ J*-V curves meauremnt instrument during thermal cycling, and the devices for measurment.


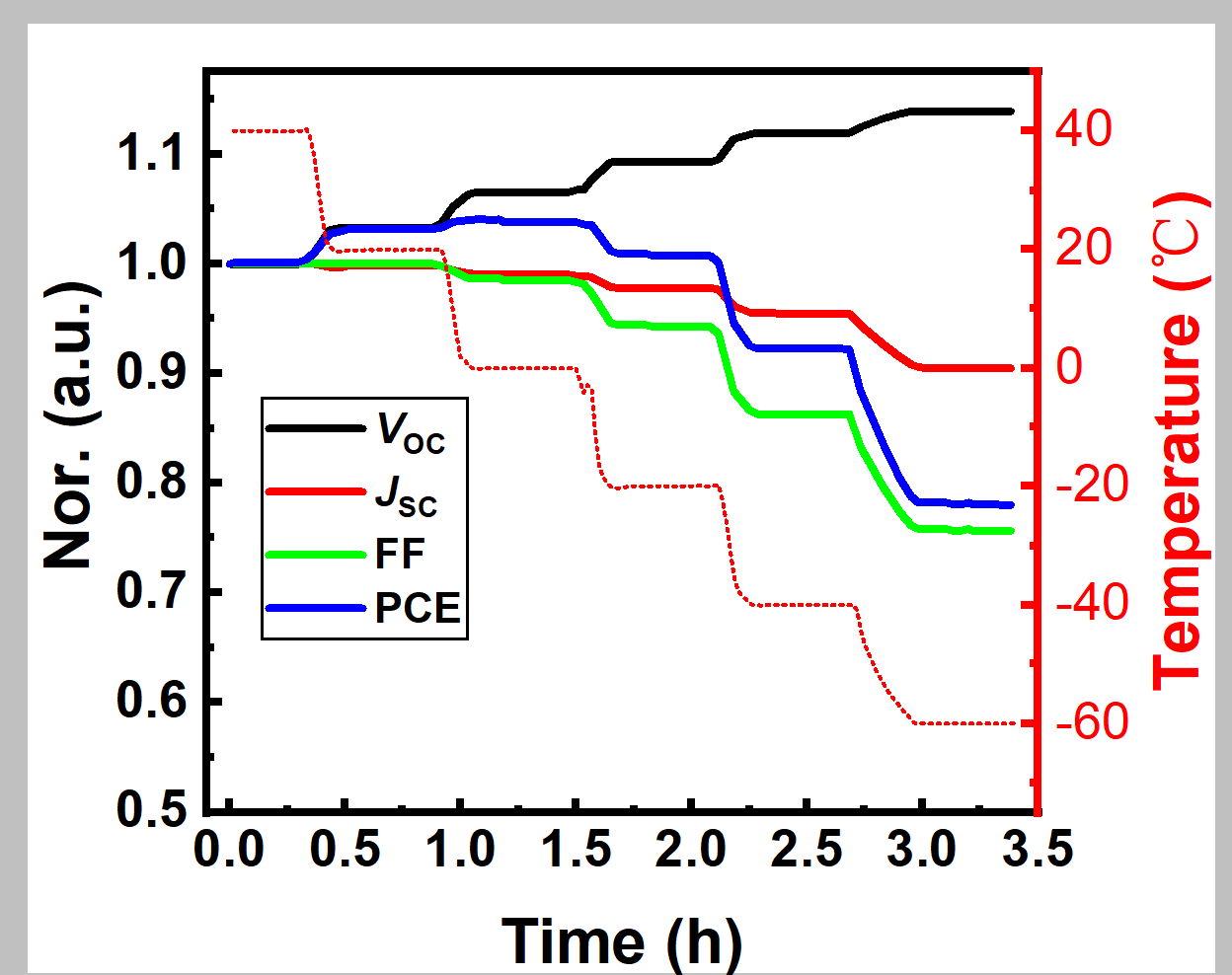


**Figure S7** Evolution of performance parameter at different temperatures (the performance was normalized at the value of 40 ^o^C).

**Figure S8** Light-intensity-dependent *J*_SC_ at different temperatures.

**Figure S9** Light-intensity-dependent *V*_OC_ at different temperatures.

**Figure S10** The electrochemical impedance spectroscopy (EIS) of the FOSCs at different temperatures.

**Figure S11** The photographs of compass that used to track the orientation of the measurement instrument.

**Figure S12** Incidence angle of the sunlight during the in-situ experiment and the variation of *J*_SC_.

**References:**

[1] Wang Z, Han Y, Yan L, *et al.* High-power conversion efficiency of 13.61% for 1 cm^2^ flexible polymer solar cells based on patternable and mass‐producible gravure‐printed silver nanowire electrodes. *Adv Funct Mater* 2020; 31: 2007276.

[2] Pan W, Han Y, Wang Z, *et al.* An efficiency of 14.29% and 13.08% for 1 cm^2^ and 4 cm^2^ flexible organic solar cells enabled by sol–gel ZnO and ZnO nanoparticle bilayer electron transporting layers. *J Mater Chem A* 2021; 9: 16889-16897.

[3] Cardinaletti I, Vangerven T, and Nagels S*, et al.* Organic and perovskite solar cells for space applications. *Sol Energy Mater Sol Cells* 2018; 182: 121-7.

[4] Tu Y, Xu G, and Yang X*, et al.* Mixed-cation perovskite solar cells in space. *Sci China Phys Mech* 2019; 62(7) 974221.

[5] Reb LK, Böhmer M, and Predeschly B*, et al.* Perovskite and organic solar cells on a rocket flight. *Joule* 2020; 4: 1880-92.
